# Zoonotic rat hepatitis E virus infection in pigs: farm prevalence and public health relevance

**DOI:** 10.1186/s40813-025-00450-9

**Published:** 2025-06-11

**Authors:** Javier Caballero-Gómez, Ignacio García-Bocanegra, David Cano-Terriza, María Casares-Jiménez, Saúl Jiménez-Ruiz, María A. Risalde, Lucía Ríos-Muñoz, Antonio Rivero-Juárez, Antonio Rivero

**Affiliations:** 1 CIBERINFEC, ISCIII– CIBER de Enfermedades Infecciosas, Instituto de Salud Carlos III, Madrid, España; 2 Grupo de Virología Clínica y Zoonosis, Unidad de Enfermedades Infecciosas, Instituto Maimónides de Investigación Biomédica de Córdoba (IMIBIC), Hospital Universitario Reina Sofía, Universidad de Córdoba, Córdoba, España; 3 Departamento de Sanidad Animal, Grupo de Investigación GISAZ, UIC Zoonosis y Enfermedades Emergentes ENZOEM, Universidad de Córdoba, Córdoba, España; 4 Departamento de Anatomía, Anatomía Patológica Comparadas y Toxicología, Grupo de Investigación GISAZ, UIC Zoonosis y Enfermedades Emergentes ENZOEM, Universidad de Córdoba, Córdoba, España

**Keywords:** Rocahepevirus, Rat hepatitis E virus, Pigs, Public health, Zoonoses, Emerging

## Abstract

Rat hepatitis E virus (ratHEV) is an emerging zoonotic pathogen causing acute and chronic hepatitis, with zoonotic transmission pathways that remain unclear. While rats are its primary reservoir, recent experimental studies revealed that pigs are susceptible to ratHEV infection. To better understand its epidemiology, we assessed the herd prevalence of ratHEV in 64 extensively managed pig farms in southwestern Spain. Serum samples from 1,861 animals were pooled and analyzed using a multiplex qPCR. Viral RNA was detected in 9 (1.9%) of the 466 analyzed pools, with a herd prevalence of 10.9% (7/64; 95%CI: 5.4–20.9). Positive farms were distributed across the study area, and ratHEV circulation was limited to fattening farms (17.5%) (*P =* 0.007). These findings suggest pigs could act as intermediate hosts between rats and humans, posing a public health risk. Enhanced surveillance in pig farms and pork products is crucial to assess zoonotic transmission risks.

**Supplementary Information:**

The online version contains supplementary material available at 10.1186/s40813-025-00450-9.

## Clinical trial number

Not applicable.

## Introduction

*Rocahepevirus ratti* (family *Hepeviridae*), also known as rat hepatitis E virus (ratHEV), is an emerging zoonotic virus of increasing public health concern [1]. Since the first infection in human was confirmed in 2018, a growing number of cases of acute and chronic hepatitis due to ratHEV have been reported globally [2–7]. Although rats are the main reservoir of the virus, its transmission pathway to humans remains poorly understood [8]. Several studies have suggested that humans may acquire the infection through contact with surfaces contaminated by rat droppings or through contaminated water or food products [2, 3, 8]. In this context, the potential role of intermediate hosts in the epidemiological cycle of ratHEV between rats and humans remains an open question.

In 2023, ratHEV RNA was detected in pig feces from two farms of Southern Spain [9]. This finding confirmed ratHEV circulation in pig farms; however, this finding did not conclusively demonstrate active infection in the host [10]. Shortly after, Yadav et al. [11] experimentally demonstrated that pigs were susceptible to ratHEV infection, identifying viral RNA in sera, different tissues, faeces and urine following challenge. In this context, survey studies assessing the burden of ratHEV in pig farms are needed to better understand the epidemiology of this emerging virus and to assess the risk to public health. Here, we aimed to assess the herd prevalence of ratHEV in pig farms managed under extensive production systems in southwestern Spain.

## Methods

### Study design and sampling

A cross-sectional study was carried out in Iberian pig farms from southwestern Spain, an important agricultural area that accounts with about 80% of the Spanish extensively raised pig populations [12]. The sample size was calculated to be 62 farms, assuming a herd prevalence of 10%, with a 95% confidence interval (CI) and an accepted error of 7.5% [9, 13]. Within each farm, at least 5% of the pig population was sampled. Sampled pigs were selected using systematic sampling: the first animal was randomly selected, and subsequent pigs were selected using a fixed sampling interval.

Blood samples from pigs were collected between 2015 and 2017 in sterile tubes without anticoagulants through the orbital sinus puncture method. No blood samples were collected specifically for this study, but were taken during routine procedures carried out by professional staff from the Regional Government of Andalusia. Ethical approval was not therefore deemed necessary. Sera were obtained after blood centrifugation at 400*g* for 10 min and frozen at -20ºC until molecular analyses. During sampling, an epidemiological questionnaire related to the animals sampled, the management and production parameters, as well as environmental data and biosecurity measures implemented on each farm was conducted through a direct interview with each farmer, who verbally consent to the animals participation in the study.

### Molecular pool analyses

Pools of 400 µL of serum samples from four pigs (100 µL each) from the same farm were prepared. RNA was extracted from pools using the total RNA Purification Kit (Norgen Biotek, Ontario, Canada) and an automated procedure (QIAcube^®^, QIAgen, Hilden, Germany). The purified RNA was eluted in a total elution volume of 50 µL, stored at -80 °C immediately after extraction and analyzed within 7 to 10 days. We have now added this information to the Methods section. The presence of ratHEV RNA was determined by multiplex RT-qPCR using the One Step PrimeScript III RT-PCR Kit and the primers and probes previously described [14, 15] in a CFX Connect Real-Time PCR Detection System (Biorad, Hercules, USA). Positive pools were further analyzed for phylogenetic analyses. Two different nested RT-PCR, targeting a conserved region of the ORF-1 of ratHEV, were carried out as previously described [4, 9]. Consensus sequences were obtained using SeqMan Software NGen^®^ Version 12.0 (DNASTAR. Madison, WI, USA) and ratHEV assignments were performed using the HEVnet genotyping tool (https://www.rivm.nl/mpf/typingtool/hev/) [16] and confirmed using the Basic Local Alignment Search Tool (BLASTn). A phylogenetic tree was constructed by the neighbour joining method using the MEGA software (Version 11) and the bootstrap method (with 1,000 replicates). For that we used complete ratHEV sequences from Wu et al. [17] as well as representative sequences previously obtained in animals and patients from Spain. Additional information about molecular analyses is shown in the Appendix.

### Statistical analyses

The outcome variable was the detection of infected pig farms, which was defined as the identification of ratHEV RNA in at least one pool of sera by either of the two qPCR methods from pigs of that farm. The herd prevalence was calculated as the proportion of positive farms and the total number of farms examined, with a 95%CI. A bivariate chi-square and Fisher’s exact test were performed to obtain the statistical significance of explanatory variables collected using the farm questionnaire with respect to the ratHEV status (positive or negative to ratHEV) at farm-level (dependent variable). Cut-off points for continuous variables were determined at the 33rd and 66th percentiles. Variables with *P-*value < 0.05 in were considered statistically significant. SPSS 22.0 software (IBM Corp., Armonk, NY, USA) was used for statistical analyses.

## Results

### Study cohort

**Fig. 1:**
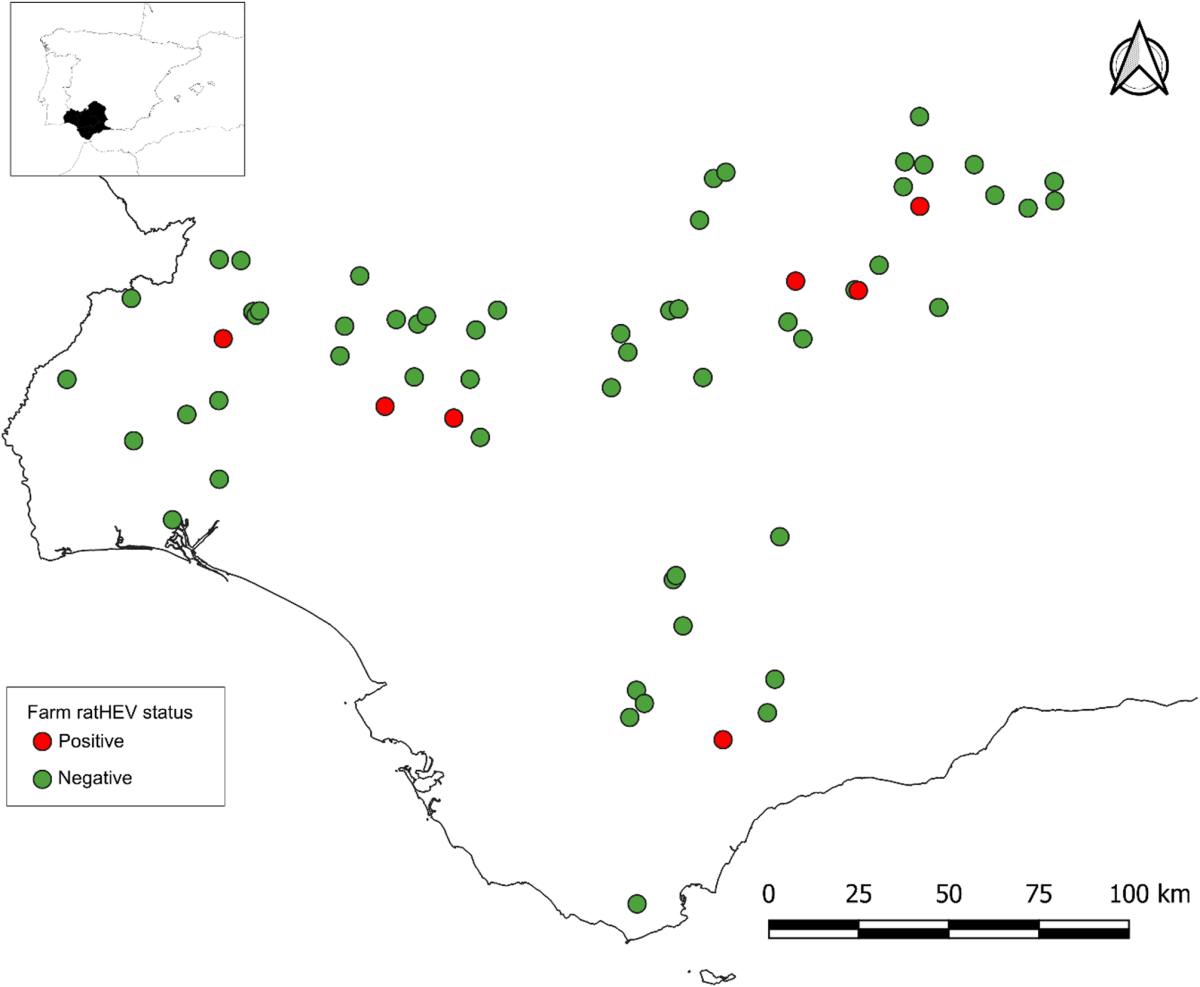
Spatial distribution and ratHEV status of sampled pig farms in southwestern Spain

The study cohort was comprised by 64 different farms, in which 1,872 Iberian pigs were sampled (Fig. 1). All farms sampled were managed under extensive production systems and 40.7% of them reported ongoing rodent control programs. With respect to their purpose, 62.5% of the farms were for fattening and 37.5% for breeding. Detailed information about the study cohort is included in Table 1.

**Table 1:** Demographic characteristics of the pig farm study population

Variables	Categories	No.*	%
Aptitude	Fattening	40	62.5
	Reproductive	24	37.5
Herd size	< 137 pigs	20	32.3
	137–342 pigs	21	33.9
	> 342 pigs	21	33.9
Restocking	Own	13	26.0
	External	19	38.0
	Mixed	18	36.0
Mixed farms with sheep	No	44	71.0
	Yes	18	29.0
Mixed farms with goat	No	52	85.2
	Yes	9	14.8
Mixed farms with cattle	No	26	41.9
	Yes	36	58.1
Presence of rodents	No	14	25.5
	Yes	41	74.5
Presence of dogs	No	13	21.3
	Yes	48	78.7
Presence of cats	No	35	57.4
	Yes	26	42.6
Presence of wild boar	No	7	12.3
	Yes	50	87.7
Presence of red deer	No	17	29.8
	Yes	40	70.2

*Missing values omitted

### Screening for RatHEV

Nine pools were positive to ratHEV by RT-qPCR, indicating a frequency of positive pools of 1.9% (9/468). The herd prevalence was 10.9% (7/64; 95%CI: 5.4–20.9) (Fig. 1), with three positive pools being obtained from the same farm, while each of the remaining pools were from different herds. Positive farms were detected throughout the study area (Fig. 1). Statistically significant differences were only observed among the production stage of pigs (*P =* 0.007). In this respect, circulation of ratHEV was detected in fattening farms (17.5%; 7/40), but not in breeding farms (0.0%; 0/24). None of the remaining variables were associated with ratHEV positivity (Table 2).

**Table 2:** Comparison of the characteristics of the pig farm study population regarding RatHEV status

Variables	Categories	RatHEV status		*P value*
		Number (%) of infected farms*	Number (%) of non-infected farms*	
Aptitude	Fattening	7 (100)	33 (57.9)	0.030
	Reproductive	0 (0)	24 (42.1)	
Herd size	< 137 pigs	3 (42.9)	17 (30.9)	0.507
	137–342 pigs	3 (42.9)	18 (32.7)	
	> 342 pigs	1 (14.2)	20 (36.4)	
Restocking	Own	2 (50.0)	17 (37.0)	0.260
	External	2 (50.0)	11 (23.9)	
	Mixed	0	18 (39.1)	
Mixed farms with sheep	No	4 (57.1)	40 (72.7)	0.326
	Yes	3 (42.9)	15 (27.3)	
Mixed farms with goat	No	7 (100)	45 (83.3)	0.307
	Yes	0 (0)	9 (16.7)	
Mixed farms with cattle	No	5 (71.4)	21 (38.2)	0.102
	Yes	2 (28.6)	34 (61.8)	
Presence of rodents	No	2 (40.0)	12 (24.0)	0.377
	Yes	3 (60.0)	38 (76.0)	
Presence of dogs	No	2 (33.3)	11 (20.0)	0.378
	Yes	4 (66.7)	44 (80.0)	
Presence of cats	No	5 (83.3)	30 (54.5)	0.181
	Yes	1 (16.7)	25 (45.5)	
Presence of wild boar	No	0 (0)	7 (13.7)	0.438
	Yes	6 (100)	44 (86.3)	
Presence of red deer	No	2 (33.3)	15 (29.4)	0.586
	Yes	4 (66.7)	36 (70.6)	

*Missing values omitted

**Fig. 2:**
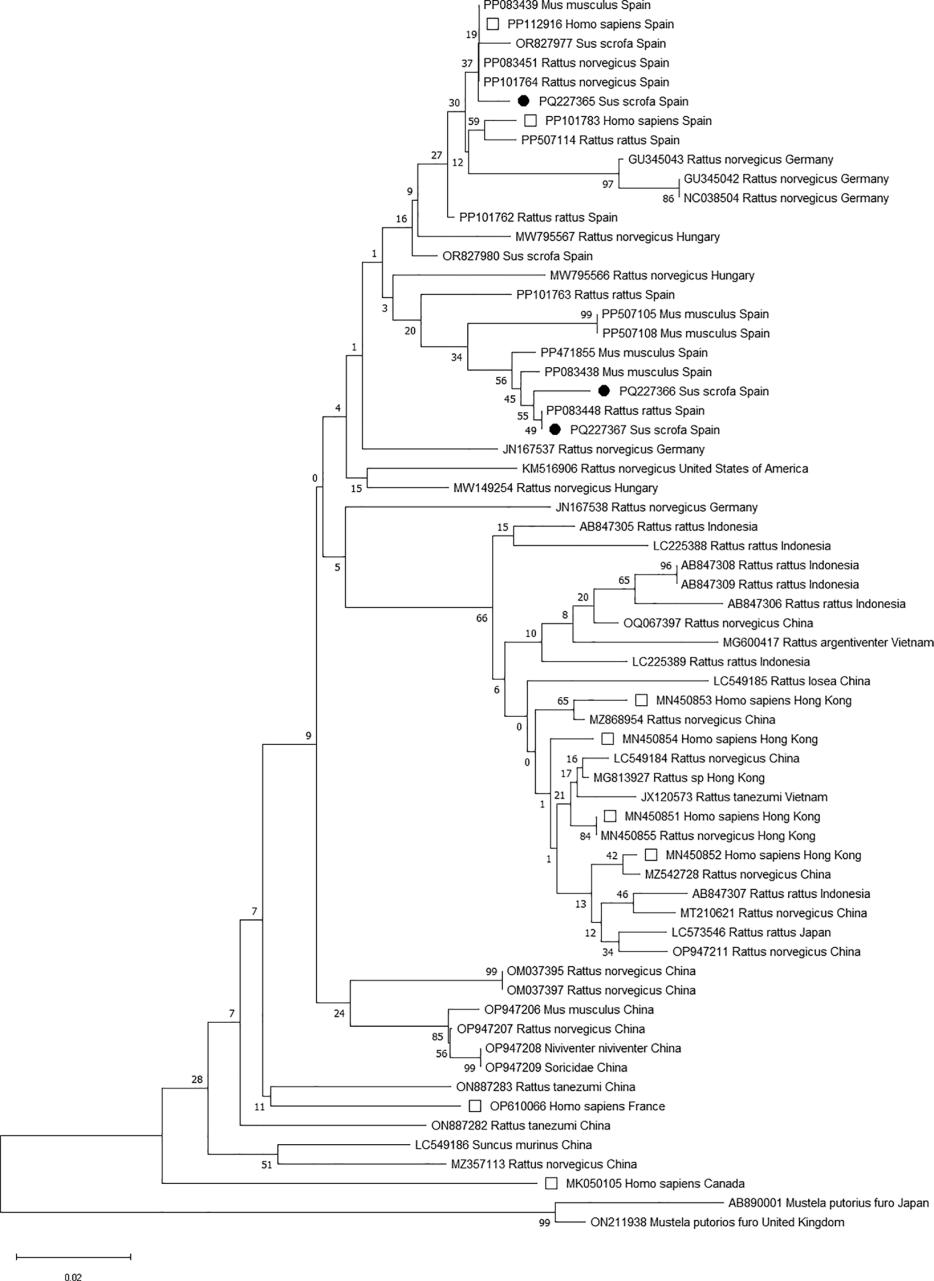
Phylogenetic tree constructed by the Neighbor-Joining method with the bootstrap test (1000 replicates) using MEGA11. The percentage of replicate trees in which the associated taxa clustered together is shown next to the branches. The tree is drawn to scale, with branch lengths in the same units as those of the evolutionary distances used to infer the phylogenetic tree. The evolutionary distances were computed using the Maximum Composite Likelihood method and are in the units of the number of base substitutions per site. Sequences obtained in the present study are presented with black circles, while white squares are used for ratHEV strains retrieved from humans. This analysis involved 64 nucleotide sequences, including complete ratHEV sequences from Wu et al. [17] as well as representative sequences from Spain, and 177 positions in the final dataset

Viral sequences were obtained in three pools, each of these obtained from different farms (GenBank Accession Numbers: PQ227365-PQ227367). The phylogenetic tree constructed clustered our sequences in two different groups phylogenetically related with other ratHEV strains obtained from animals from Spain, Germany and Hungary and from patients suffering acute hepatitis from Spain (Fig. 2).

## Discussion

RatHEV is a newly identified zoonotic and emerging virus, the epidemiology of which, particularly its transmission route, remains poorly understood. Remarkably, although circulation of ratHEV has been detected globally in rodents, almost none of the human cases reported so far show any direct epidemiological link with these species [1, 15]. In this context, identifying potential reservoir species beyond rodents that could act as source of ratHEV transmission to humans is crucial for elucidating transmission routes and developing targeted control measures against this emerging virus. Here, we provide evidence that pigs may represent a public health concern for ratHEV transmission. The proportion and spatial distribution of positive herds suggest that ratHEV is locally widespread among extensively raised pig populations in the study area. This finding aligns with the high prevalence recently observed in pest rats (29.8%) from Spanish farms [18] which, together with the high homology of ratHEV strains found in pigs and rats evidenced in our study, point to natural cross-species transmission of the virus between these species. Furthermore, rats are frequently found on pig farms worldwide and circulation of ratHEV has already been detected in rats surrounding pig farms from Asia and other countries of Europe [19–23]suggesting that ratHEV infection in pigs may be frequent.

Experimental studies have also evidenced that the virus is mainly shed by urine and faeces of infected pigs and that fecal-oral transmission of ratHEV occurs among co-housed pigs [11] highlighting the potential for ratHEV transmission within the same farm. While repeated transmission of the virus from rats to pigs cannot be ruled out, natural intraspecific transmission of the virus is supported by the detection of three positive pools from the same pig farm in the present study as well as by the high frequency of positivity (58.6%) recently detected in pig faeces from one intensive pig farm in the study area [9]. Likewise, ratHEV transmission may also occur not only between pigs but also from pigs to other animal species [24]. It should be noted that extensively raised pigs share habitats and frequently contact with other domestic and wildlife species [25]. In this sense, all ratHEV-positive herds reported frequently contact between pigs and other domestic or wild species. Considering the expanding host range of HEV, a preliminary evaluation of ratHEV in closely sympatric animal species should also be conducted.

The detection of natural ratHEV infection in pigs supports the hypothesis that this species could be a source of ratHEV transmission for humans. In industrialized countries, foodborne transmission is the primary zoonotic route of infection for the closely related HEV [26]. Thus, several studies have documented cases of human HEV infections linked to the consumption of raw or undercooked meat, liver, or liver sausages derived from pigs [27, 28]. Remarkably, Yadav et al. [11] recently demostrated the presence of the virus in the liver of viremic pigs after challenging with ratHEV strains. In the present study, the detection of viral RNA in sera from Iberian pigs, and particularly in fattening pigs, suggests that the presence of ratHEV in pork products is likely plausible. Further research in pork products is warranted to determine the presence and viability of ratHEV in order to assess the risk of zoonotic transmission. In this regard, the major pork products from the Iberian pig—an endemic breed of the Iberian Peninsula— and its crossbreeds include not only meat but also cured products such as ham, *salchichon*, or *chorizo* sausages, which are consumed raw or undercooked.

This study has several limitations that should be acknowledged. First, all farms included in this study were managed under extensive production systems. Although highly relevant from ecological and high-quality production standpoints, they are less represented in the overall pig production landscape in Europe, where intensive systems predominate. Therefore, the findings may not be generalizable to other farming contexts, particularly those with different biosecurity standards and animal densities. Second, sera analyzed in this study were collected between 2015 and 2017, before the virus was first detected in humans, which could limit the interpretation of the findings in the current epidemiological context. Nevertheless, these samples provide valuable retrospective evidence of early ratHEV circulation in pigs. Lastly, the lack of sequence data from all positive pools constrained the phylogenetic analysis and may have led to an underestimation of viral diversity within and among farms.

In conclusion, the detection of ratHEV RNA in pig sera, along with the phylogenetic similarity of the strains found in our study with those previously identified in rodents and human cases, underscores the potential role of pigs as intermediary hosts in the zoonotic transmission cycle of ratHEV. These findings highlight the need for enhanced surveillance in pig farms and pork products to better understand the risk of zoonotic transmission from this species. Such efforts will be essential for developing effective control strategies against this emerging public health threat.

## Electronic supplementary material

Below is the link to the electronic supplementary material.

Supplementary Material 1

## Data Availability

All of the data generated or analyzed during the study are included in the article. The datasets used and/or analyzed during the present research project are available from the corresponding author upon reasonable request to minimize privacy risks and concerns for farmers. The viral sequences are available in GenBank under accession numbers PQ227365-PQ227367 (https://www.ncbi.nlm.nih.gov/nuccore/?term=PQ227365; https://www.ncbi.nlm.nih.gov/nuccore/?term=PQ227366; https://www.ncbi.nlm.nih.gov/nuccore/?term=PQ227367).
