# Supplementary material for: Zoonotic rat hepatitis E virus infection in pigs: farm prevalence and public health relevance
Source: Porcine Health Manag. 2025 Jun 11;11:34. doi: 10.1186/s40813-025-00450-9 (PMC12160372; doi:10.1186/s40813-025-00450-9)
Supplement: Supplementary file 1 — Supplementary Material 1 [file 40813_2025_450_MOESM1_ESM.docx]

**Appendix**

The primers and probes for RT-qPCR is shown in Table S1. For each reaction, 20µl of RNA, 20µl of master mix, 5.5µL of RNase-DNase free water, 0.75µL of each primer (20µM) and 0.75µL of each probe (20µM) was added. The thermal profile was 25ºC for 10 min, 52ºC for 5 min and 95ºC for 10 sec, followed by 45 cycles of 95ºC for 5 sec and 56ºC for 30 sec. For phylogenetic analyses, two nested RT-PCRs were carried out using the primers indicated in Table S1. As a positive control, we used RNA from a rodent liver sample previously tested as ratHEV positive in our laboratory (GenBank accession number: OR282813). **As negative control, we used nuclease free water. Both positive and negative controls were used in each run of qPCR**.

| **PCR** | **Ref** | **PCR product (pb) (position*)** | **Primers (5'-3')** | | **Probe (5'-3')** |
| --- | --- | --- | --- | --- | --- |
|  |  |  | **Forward** | **Reverse** |  |
| RT-qPCR | ^1^ | 69 (146-214) | CTTGTTGAGCTYTTCTCCCCT | CTGTACCGGATGCGACCAA | HEX- TGCAGCTTGTCTTTGARCCC -IABkFQ |
|  | ^2^ | 173 (36-208) | CCACGGGGGTTAATACTGC | CGGATGCGACCAAGAAACAG | 6FAM-CGGCTACCG/ZEN/CCTTTGCTAATGC/3IABkFQ |
| Nested RT-PCR | ^3^ | 287 (164-450) | 1^st^ PCR: TTTGCTAATGCTCAGGTGGT | 1^st^ PCR: CATDCCATGAGCACGCAT |  |
|  |  |  | 2^nd^ PCR: CCTYTGCAGCTTGTCTTTGA | 2^nd^ PCR: GTGCAAAAGGAAAGATCAG |  |
|  | ^4^ | 243 (189-450) | 1^st^ PCR: CCTYTGCAGCTTGTCTTTGA | 1^st^ PCR: CATDCCATGAGCACGCAT |  |
|  |  |  | 2^nd^ PCR: CTGTTTCTTGGTCGCATCCG | 2^nd^ PCR: GTGCAAAAGGAAAGATCAG |  |
|  | *Genome positions respect to the reference RHEV sequence under the GenBank Accession Number GU345042 | | | | |

**Table S1**. List of primers and probes, when applicable, of molecular assays used in the present study.

**References**

1. Suparyatmo, J. B., Andayani, I. G. A. S., Takahashi, M., Ohnishi, H., Jirintai, S., Nagashima, S., ... & Okamoto, H. (2014). Marked genomic heterogeneity of rat hepatitis E virus strains in Indonesia demonstrated on a full-length genome analysis. *Virus research*, *179*, 102-112.
2. Sridhar, S., Yip, C. C., Wu, S., Cai, J., Zhang, A. J. X., Leung, K. H., ... & Yuen, K. Y. (2018). Rat hepatitis E virus as cause of persistent hepatitis after liver transplant. *Emerging infectious diseases*, *24*(12), 2241.
3. Caballero-Gómez, J., Fajardo-Alonso, T., Ríos-Muñoz, L., Beato-Benítez, A., Casares-Jiménez, M., García-Bocanegra, I., … & Rivero, A. National survey of the rat hepatitis E virus in rodents in Spain, 2022-2023. *Eurosurveillance*, in press.
4. Caballero-Gómez, J., Pereira, S., Rivero-Calle, I., Perez, A. B., Viciana, I., Casares-Jiménez, M., ... & Rivero, A. (2024). Acute Hepatitis in Children Due to Rat Hepatitis E Virus. *The Journal of Pediatrics*, 114125.
